# Effects of dietary supplementation with a thymol-carvacrol blend on growth performance and intestinal health of poultry

**DOI:** 10.3389/fvets.2025.1739666

**Published:** 2026-01-12

**Authors:** Xiaoxia Liu, Xiang Li, Ruiying Chen, Jing Liu, Rui Liu, Ruting Zhao, Aiguo Luo, Jia Zhao, Jianwei Hao, Shuming Yang, Ailiang Chen

**Affiliations:** 1 Department of Biological Science and Technology, Shanxi Center of Technology Innovation for Compound Condiment, Jinzhong University, Jinzhong, China; 2 Key Laboratory of Agro-product Quality and Safety, Institute of Quality Standard and Testing Technology for Agro-Products, Chinese Academy of Agricultural Sciences, Beijing, China; 3 School of Food Science and Engineering, Shanxi Agricultural University, Jinzhong, China; 4 School of Investigation, People's Public Security University of China, Beijing, China

**Keywords:** antibiotics, carvacrol, gut microbiota, plant essential oils, thymol

## Abstract

**Background:**

Antibiotic resistance has intensified the search for alternatives in poultry production. Essential oils (EOs), particularly blends of carvacrol and thymol, have shown potential as natural growth promoters and antimicrobials. This study evaluated a composite carvacrol-thymol EO as an antibiotic substitute in broiler production, focusing on growth performance, serum biochemistry, intestinal morphology, and gut microbiota.

**Materials and methods:**

A total of 672 Aibayi-Yijia broilers were randomly assigned to seven treatment groups: control (CK), EO1 (200 g/t feed), EO2 (600 g/t feed), EO3 (1200 g/t feed), EO1+AG (EO 200 g/t + FON 0.15 g/kg feed), EO3+AG (EO 1200 g/t + FON 0.15 g/kg feed), and AG (FON 0.15 g/kg feed). Growth performance, serum biochemistry (TP, ALB, GLB, GLU, AST, GGT, CHOL, TG, IL), jejunal histology (villus height, crypt depth, V:C ratio), and cecal microbiota (16S rRNA sequencing) were assessed.

**Results:**

Supplementation with EO (600 g/t) or florfenicol followed by 1200 g/t EO significantly increased ABW and ADFI (*p* < 0.05). EO (200 g/t or 1200 g/t) supplementation after antibiotics reduced serum TP, ALB, GLB, and CHOL (*p* < 0.05). Histological analysis showed increased villus height and V:C ratios with 1200 g/t EO. Cecal microbiota shifted, with increased Bacteroidetes and decreased Firmicutes.

**Discussion:**

The composite carvacrol-thymol EO blend showed promise as an antibiotic alternative, improving growth performance, supporting intestinal health, and modulating the gut microbiota. Further research is needed to optimize dosing, assess long-term safety, and explore EO interactions for scalable use.

## 1 Introduction

Intensification of livestock production has increased demands for faster growth, improved feed conversion efficiency, and higher meat quality in broiler chickens (1). In broiler production, antibiotics are commonly added to feed to enhance growth and prevent disease (2). However, indiscriminate use has promoted antimicrobial resistance and generated drug residues. It has also caused environmental contamination, thereby threatening animal health and food safety (3, 4). Recent national regulations have restricted or banned antibiotic growth promoters in feed. Consequently, researchers and producers are pursuing environmentally friendly, effective alternatives.

Plant essential oils (EOs) are volatile, bioactive phytochemicals proposed as alternatives to antibiotics because of their antibacterial, anti-inflammatory, antioxidant and digestion promoting properties (5, 6). Wińska and colleagues reviewed the antibacterial activities and mechanisms of multiple EOs and their constituents (7). Vlaicu et al. summarized experimental evidence that EOs affect digestion, intestinal morphology, immunity and growth performance, and discussed their potential as antibiotic substitutes (6, 8). In particular, the monoterpenoid phenols carvacrol and thymol exhibit pronounced antimicrobial activity, modulate the gut microbiota and improve intestinal health, thereby contributing to enhanced feed efficiency and growth in poultry (9, 10). Several reports indicate that a thymol-carvacrol eutectic markedly improves intestinal morphology and barrier function and significantly enhances growth and health in broilers (11, 12). EO efficacy is influenced by inclusion level, component ratio and timing of administration. Consequently, the precise mechanisms and optimal application protocols required to replace antibiotics remain to be determined.

Arbor Acres Plus broilers were used to evaluate a composite essential oil (carvacrol + thymol) as an alternative to antibiotics. Different inclusion levels and feeding schedules of the blend were compared with a conventional florfenicol regimen. Effects on production performance (ABW, ADG, ADFI, F/G) and serum biochemistry (TP, ALB, GLB, CHOL) were assessed. Small intestinal histomorphology (villus height, crypt depth, V: C) and cecal microbiota (16S rRNA) were also examined. The study aimed to provide theoretical support and practical guidance for the use of plant essential oils in antibiotic free broiler diets.

## 2 Materials and methods

### 2.1 Experimental materials

Male day-old Arbor Acres Plus (AA) broiler chicks were obtained from Beijing Dafa Chia Tai Co., Ltd. (also listed as Beijing Dafa Zhengda Co., Ltd.). The essential oil feed additive (total effective essential-oil content ≥3.5%; carvacrol ≥2.3%; thymol ≥1.2%) was supplied by Guangzhou Wisdom Bio-Technology Co., Ltd. (also listed as Guangzhou Zhiteqi Bio-technology Co., Ltd.). Florfenicol soluble powder (30% w/w) was purchased from Henan Muxiang Biological Co., Ltd. (Henan Muxiang Animal Pharmaceutical/Biotech).

### 2.2 Experimental design and grouping

A total of 700 one-day-old male Arbor Acres (AA) broiler (initial body weight, 45.75 ± 0.30 g) were selected and housed in cages under continuous lighting. The house temperature was maintained at 34 ± 1 °C for 3 days before broiler placement; comprehensive disinfection was performed prior to arrival. Thereafter, temperature was reduced by 2 °C per week until it reached 24 °C, and this temperature was kept until the end of the trial. Feed and water were provided ad libitum throughout the experiment. Ventilation, hygiene and manure removal were routinely maintained, and regular disinfection was performed. Diets were formulated for two phases: starter (1–21 d) and grower (22–42 d). Basal diets met the nutrient recommendations of NRC (1994) and the Chinese feeding standard NY/T 33-2004. Dietary amino-acid balance was achieved using an ideal digestible amino-acid profile. The ingredient composition and nutrient levels of the experimental diets are presented in Table 1.

**Table 1:** Ingredients and nutrient composition of diets.

Component	1 ~ 21 d (Level/%)	22 ~ 42 d (Level/%)
Ingredient component		
Corn	48.8834	53.08
Soybean meal	38.7448	33.4638
Soybean oil	3.6825	4.704
Wheat middling	5	5
Limestone	1.05	1.05
NaCl	0.1385	0.1326
Sodium bicarbonate	0.2395	0.2502
Choline chloride	0.1	0.1
Dicalcium P	1.6214	1.6954
L-lysine	0.0869	0.1084
DL-methionine	0.2767	0.2424
Threonine	0.0562	0.0533
Vitamin Premix^1^	0.02	0.02
Mineral Premix^2^	0.1	0.1
Total	100	100
Nutrient component^3^		
CP%	22	20
Energy	3,000	3,100
Lys%	1.12	1.02
Methionine cystine	0.76	0.76
Threonine	0.67	0.67
Tryptophan	0.2036	0.1993
Valine	0.8201	0.8168
Leucine	1.4454	1.4712
Isoleucine	0.7324	0.7255
Arginine	1.2502	1.2253
Met%	0.5048	0.5002
Histidine	0.4737	0.4748
Glycine Serine	1.7128	1.6931
Tryptophan	0.2036	0.1993
Lys + Met	1.5048	1.5002
Potassium	0.9745	0.8789
Sodium	0.175	0.175
Chlorine	0.175	0.175
Calcium	0.89	0.89

1 Vitamin premix provided per kg of diet: VA 12500 IU; VB₁ 2.0 mg; VB₂ 6.0 mg; VB₆ 3.0 mg; VB₁₂ 0.025 mg; VD₃ 2,500 IU; VK₃ 2.65 mg; VE 50 mg; pantothenic acid 10.5 mg; niacin 50 mg; biotin 0.0325 mg; folic acid 0.25 mg.

2 Mineral premix provided per kg of diet: Zn 75 mg; Mn 100 mg; Cu 8 mg; Fe 80 mg; Se 0.15 mg; I 0.35 mg.

3 All nutrient levels were calculated values.

The EO inclusion levels were selected with reference to the dose response patterns reported by Yang et al. (13) in broilers receiving thymol–carvacrol cocrystal and by Hong et al. (14), who demonstrated clear graded effects of encapsulated essential oils in meat ducks. Based on this evidence, EO was included at 200, 600, and 1,200 g/t to cover a biologically relevant response range. Florfenicol was included at 0.15 g/kg, a dosage commonly applied in poultry practice and widely used as a reference level in comparative studies on antibiotic alternatives. To further assess potential EO antibiotic interactions, two combination treatments were added: low-dose EO + florfenicol and high-dose EO + florfenicol.

At the start of the trial, AA broiler chicks were randomly assigned to one control group and six treatment groups. Each treatment included eight replicates of 12 birds per replicate, resulting in 672 birds after day-one sampling and culling. Grouping details are shown in Table 2.

**Table 2:** Experimental design.

Group	Dosage level	Number of days of breeding	Distribution of samples
CK, A (Control group, Feed)	Basic diet	0–42 days (all)	96 (8 replicates, 12 each replicate)
EO1, B (Low-dose essential oil group, Feed)	EO1 200 g/t	0–42 days (all)	
EO2, C (Medium-dose essential oil group, Feed)	EO2 600 g/t	0–42 days (all)	
EO3, D (High-dose essential oil group, Feed)	EO3 1,200 g/t	0–42 days (all)	
EO1+AG, E(EO1+Florfenicol)	EO1 200 g/t + AG 0.15 g/kg	Antibiotics (days 7–21), Feed B for remaining days	
EO3+AG, F(EO3+Florfenicol)	EO3 1,200 g/t + AG 0.15 g/kg	Antibiotics (days 7–21), Feed D for remaining days	
AG, G (Florfenicol)	AG (0.15 g/kg)	Antibiotics (days 7–21), Feed A for remaining days	

### 2.3 Determination of indicators and methods

#### 2.3.1 Measurement of growth performance of AA broiler chicks

Growth performance was monitored throughout the experiment. Birds were fasted for 12 h prior to weighing. Fasted body weights (BW) were recorded at 1, 21 and 42 d of age, and the weights of sampled birds were noted. Performance parameters were calculated for each replicate and comprised average body weight (ABW), average daily gain (ADG), average daily feed intake (ADFI), feed: gain ratio (F/G) and mortality rate (MER). Calculations were performed as follows:


$$\mathrm{ADG}(g)=\frac{G_{2}-G_{1}+G_{3}}{A_{1}\times T_{1}+B_{1}\times T_{1}}$$ (1)

In Equation (1), the variables were defined as follows: G1 was the initial pen weight (g). G2 was the final pen weight (g). G3 was the combined weight of birds that died or were culled at removal (g). A1 was the number of birds remaining per cage (birds). T1 was the total experimental duration (days, d). B1 was the number of birds that died or were culled (birds).


$$\text{ADFI}(g)=\frac{G_{4}-G_{5}}{A_{1}\times T_{1}+B_{1}\times T_{1}}$$ (2)

In Equation (2), the variables were defined as follows: G4 was the total amount of feed supplied (g). G5 was the feed remaining in the trough (g). A1 was the number of birds remaining per cage (birds). T1 was the total experimental duration (days, d). B1 was the number of birds that died or were culled (birds).


$$F/G=\frac{C_{1}}{C_{2}}$$ (3)

In Equation (3), the variables were defined as follows: C1 was the average daily feed intake (g). C2 was the average daily gain (g).

#### 2.3.2 Determination of serum biochemical parameters in AA broiler chickens

Birds were sampled at 21 and 42 d of age. For each replicate, one bird close to the replicate mean body weight was selected. The wing root (brachial) vein was disinfected with 70% ethanol and blood was collected using a single use needle. Whole blood was drawn into plain vacuum tubes without anticoagulant (red top tubes). At least twice the volume required for planned serum assays was collected. Tubes were allowed to clot and were incubated in a 37 °C water bath for approximately 31 min. Samples were centrifuged at 3,500 rpm for 10 min; the pale-yellow supernatant (serum) was transferred to sterile, labeled microcentrifuge (Eppendorf) tubes and stored at −80 °Cuntil analysis.

Serum biochemical parameters were measured using an automated clinical chemistry analyzer. Assays included *γ*-glutamyl transferase (GGT), aspartate aminotransferase (AST), total protein (TP), albumin (ALB), glucose (GLU), triglycerides (TG), total cholesterol (CHOL) and globulin (GLB). All clinical biochemistry tests were performed at the Diagnostic Center of the Veterinary Teaching Hospital, China Agricultural University.

Serum concentrations of interleukin-6 (IL-6) and interleukin-10 (IL-10) were quantified by ELISA. A multifunctional microplate reader (Tecan) was used for absorbance measurements. Commercial ELISA kits were obtained from Shanghai Jianglai Biotech (Shanghai Jianglai Biological Technology Co., Ltd.).

#### 2.3.3 Determination of villus height and crypt depth in the small intestine of AA broiler chickens

At 42 d of age, 56 AA broilers were slaughtered. Approximately 2 cm segments of the duodenum, jejunum and ileum were excised, gently flushed with phosphate buffered saline (PBS) to remove luminal contents, and fixed in 4% paraformaldehyde. Tissues were processed for paraffin embedding using standard procedures (trimming, dehydration, clearing, infiltration and embedding), sectioned and stained with hematoxylin and eosin (H&E). Slides were examined with a light microscope, and intact, straight villi and their adjacent crypts were measured using Image-Pro Plus 6.0. For each section, five complete villi (red arrows in Supplementary Figure 1) and five crypts (yellow arrows in Supplementary Figure 1) were measured, and the villus height to crypt depth ratio (V: C) was calculated.

#### 2.3.4 16S rRNA gene sequencing of the gut microbiota

A total of 112 AA broilers were slaughtered by cervical dislocation at 21 and 42 days of age. Fresh cecal digesta were aseptically collected from each bird and immediately snap frozen in liquid nitrogen. Samples were stored at −80 °C until further analysis.

Microbiota quantification was carried out following the protocol of Sun et al. (46). Purified material was submitted to Biomarker Technologies Co., Ltd. (Beijing, China) for 16S rRNA gene sequencing.

### 2.4 Statistical analysis

Data were entered into Microsoft Excel and analyzed using IBM SPSS Statistics 22.0 (IBM Corp., Armonk, NY, USA). One-way analysis of variance (ANOVA) was used for comparisons among three or more groups. When ANOVA indicated a significant effect, multiple comparisons were performed using Duncan’s multiple range test or the least significant difference (LSD) post-hoc test, as appropriate. Results were reported as mean ± standard error of the mean (SEM). All tests were two tailed, and *p* < 0.05 was considered statistically significant.

## 3 Results

### 3.1 Effects of dietary compound plant essential oils on broiler growth performance

As shown in Table 3, over days 1–42 the experimental groups EO2, EO1+AG, EO3+AG and AG had significantly higher average body weight (ABW) than CK (*p* < 0.05). EO2 and EO3+AG also showed increased average daily feed intake (ADFI) (*p* < 0.05). No significant differences in average daily gain (ADG) were observed among groups (*p* > 0.05). The AG group exhibited the highest mortality rate. During days 1–21, ADG was higher in AG than in CK (*p* < 0.05). The EO1+AG group had lower ADFI than CK (*p* < 0.05). F/G was reduced in AG relative to CK (*p* < 0.05). No differences in ABW were detected among groups (*p* > 0.05). The highest mortality in this phase was observed in EO3. From days 22–42, ABW was greater in EO2, EO1+AG, EO3+AG and AG compared with CK (*p* < 0.05). ADFI was lower in EO3+AG (*p* < 0.05). No significant differences in ADG or F/G were found among groups (*p* > 0.05). The AG group again showed the highest mortality.

**Table 3:** Effects of dietary compound plant essential oils on growth performance of AA broilers.

Items	ABW/g	ADG/g	ADFI/g	F/G	MER/%
1 ~ 21 Age					
A(CK)	957 ± 0.05	42.47 ± 2.99^ab^	56.31 ± 3.28^b^	1.33 ± 0.03^b^	2.08
B(EO1)	963 ± 0.03	42.40 ± 3.25^ab^	55.74 ± 2.21^ab^	1.32 ± 0.07^ab^	4.17
C(EO2)	959 ± 0.02	43.69 ± 2.09^ab^	55.84 ± 1.06^ab^	1.28 ± 0.06^ab^	5.21
D(EO3)	942 ± 0.03	41.08 ± 2.92^a^	54.13 ± 3.40^ab^	1.32 ± 0.03^ab^	8.33
E(EO1+AG)	943 ± 0.03	42.50 ± 3.43^ab^	53.60 ± 2.49^a^	1.27 ± 0.07^ab^	4.17
F(EO3+AG)	965 ± 0.04	42.57 ± 3.87^ab^	54.21 ± 3.27^ab^	1.28 ± 0.14^ab^	1.04
G(AG)	961 ± 0.03	44.80 ± 3.93^b^	55.50 ± 2.12^ab^	1.24 ± 0.08^a^	2.08
22 ~ 42 Age					
A(CK)	2,540 ± 0.19 ^a^	61.21 ± 7.73	116.08 ± 11.56^ab^	1.91 ± 0.10	9.38
B(EO1)	2,663 ± 0.08 ^ab^	61.80 ± 4.63	113.05 ± 5.87^ab^	1.83 ± 0.12	11.5
C(EO2)	2,728 ± 0.15 ^b^	65.08 ± 12.81	119.35 ± 11.17^ab^	1.90 ± 0.39	6.25
D(EO3)	2,606 ± 0.37 ^ab^	60.49 ± 9.96	106.30 ± 19.22^a^	1.75 ± 0.07	6.25
E(EO1+AG)	2,694 ± 0.11 ^b^	64.66 ± 5.29	118.36 ± 9.03^ab^	1.84 ± 0.11	8.33
F(EO3+AG)	2,705 ± 0.19 ^b^	69.40 ± 9.93	122.45 ± 11.32^b^	1.78 ± 0.14	9.38
G(AG)	2,706 ± 0.19 ^b^	65.54 ± 14.11	116.27 ± 20.16^ab^	1.79 ± 0.17	16.7
1 ~ 42 Age					
A(CK)	60.85 ± 4.61 ^a^	51.62 ± 4.42	85.46 ± 6.46^ab^	1.66 ± 0.05^b^	11.5
B(EO1)	63.82 ± 1.79 ^ab^	51.86 ± 3.25	83.69 ± 3.43^ab^	1.62 ± 0.08^ab^	15.6
C(EO2)	65.41 ± 3.49 ^b^	54.12 ± 6.71	86.82 ± 5.75^b^	1.62 ± 0.15^ab^	11.5
D(EO3)	62.44 ± 2.48 ^ab^	50.55 ± 6.12	79.58 ± 10.50^a^	1.57 ± 0.03^a^	14.6
E(EO1+AG)	64.58 ± 2.51 ^b^	53.31 ± 2.80	85.19 ± 4.95^ab^	1.60 ± 0.07^ab^	12.5
F(EO3+AG)	64.86 ± 4.40 ^b^	55.66 ± 4.22	87.50 ± 6.34^b^	1.57 ± 0.07^a^	10.4
G(AG)	64.88 ± 4.37 ^b^	54.91 ± 6.62	85.15 ± 9.61^ab^	1.55 ± 0.08^a^	18.8

In the same column, values with the same or no letter superscripts mean no significant difference (*p* > 0.05), while with different small letter superscripts mean significant difference (*p* < 0.05).

Treatments: (A) Control (CK), basal diet; (B) EO1, essential oils 200 g/t; (C) EO2, essential oils 600 g/t; (D) EO3, essential oils 1,200 g/t; (E) EO1+AG, EO 200 g/t + florfenicol (0.15 g/kg, days 7–21); (F) EO3+AG, EO 1200 g/t + florfenicol (0.15 g/kg, days 7–21); (G) AG, florfenicol (0.15 g/kg, days 7–21).

### 3.2 Effects of dietary composite plant essential oils on serum biochemical parameters

**Figure 1:**
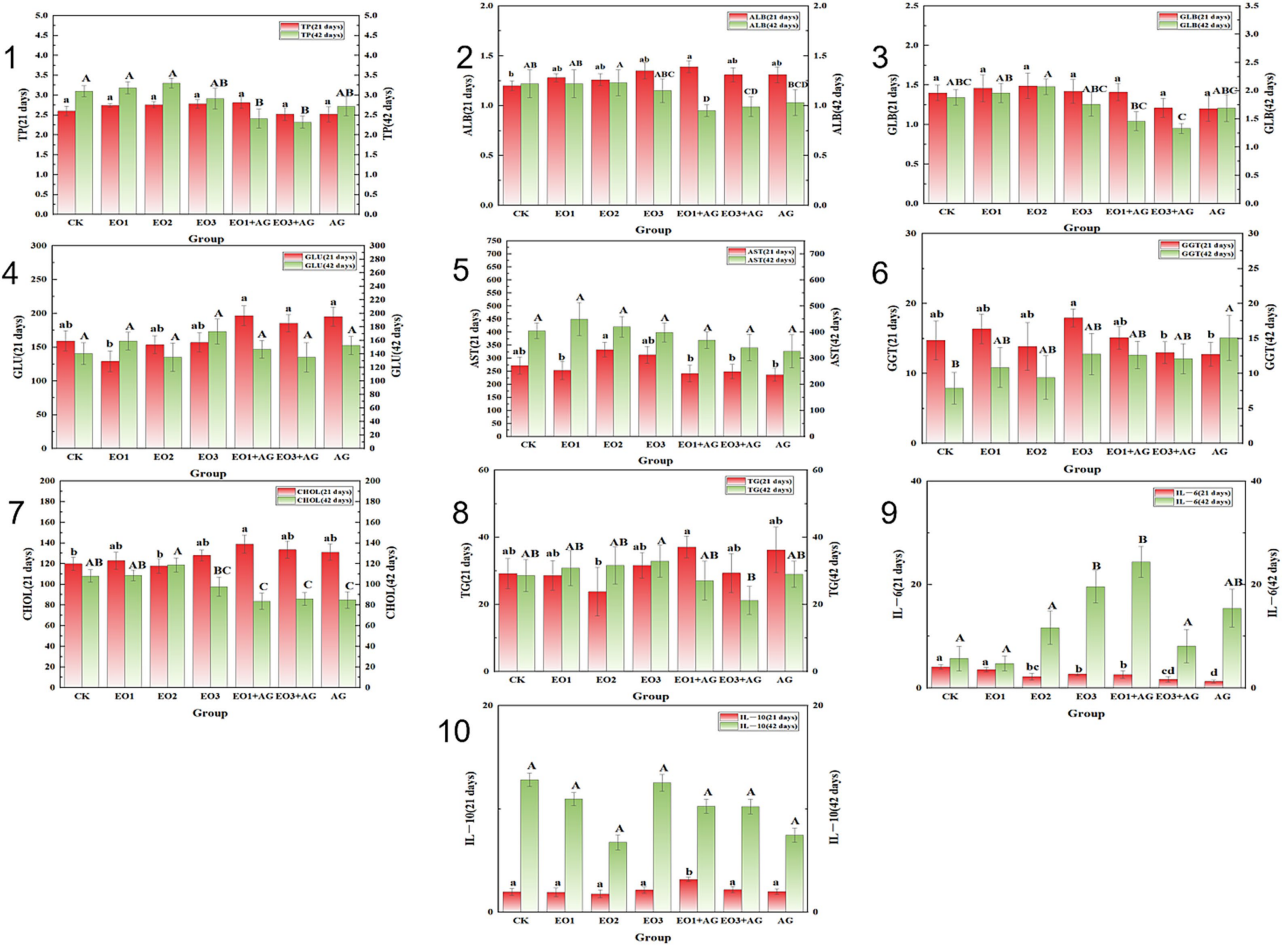
Effects of dietary compound plant essential oils on serum biochemical. Serum biochemical and immune indices of AA broilers. (1) Total protein; (2) albumin; (3) globulin; (4) glucose; (5) aspartate aminotransferase; (6) *γ*-glutamyl transferase; (7) total cholesterol; (8) triglycerides; (9) interleukin-6; (10) interleukin-10. Dietary treatments: CK, control (basal diet without antibiotics or essential oils); EO1, low EO (essential oils, 200 g/t); EO2, medium EO (essential oils, 600 g/t); EO3, high EO (essential oils, 1,200 g/t); EO1+AG, low EO + antibiotic (essential oils, 200 g/t + florfenicol, 0.15 g/kg, days 7–21); EO3+AG, high EO + antibiotic (essential oils, 1,200 g/t + florfenicol, 0.15 g/kg, days 7–21); AG, antibiotic (florfenicol, 0.15 g/kg, days 7–21). Different letters indicate significant differences among treatments (*p* < 0.05).

Figure 1 and Supplementary Table 1 summarize the serum biochemical and cytokine results. At 21d, dietary supplementation with the composite plant essential oil did not affect total protein (TP) or globulin (GLB) (*p* > 0.05). Albumin (ALB) was higher in EO1+AG than in CK (*p* < 0.05). Aspartate aminotransferase (AST) activity was increased in EO1, EO1+AG, EO3+AG and AG compared with CK (*p* < 0.05). Total cholesterol (CHOL) and interleukin-10 (IL-10) were elevated in EO1+AG versus CK (*p* < 0.05). By contrast, glucose (GLU) in EO1, gamma glutamyl transferase (GGT) in EO3+AG and AG, triglycerides (TG) in EO2, and interleukin-6 (IL-6) in EO2, EO3, EO1+AG, EO3+AG and AG were all lower than CK (*p* < 0.05).

At 42 d, no treatment effects were detected for GLU, AST or IL-10 (*p* > 0.05). TP, ALB and GLB were reduced in EO1+AG and EO3+AG compared with CK (*p* < 0.05). CHOL was lower in EO1+AG, EO3+AG and AG than in CK (*p* < 0.05), and CHOL in the antibiotic supplemented groups (EO1+AG, EO3+AG, AG) was lower than in the essential oil only groups (EO1, EO2) (*p* < 0.05). GGT was higher in AG than in CK (*p* < 0.05). IL-6 was increased in EO1+AG relative to CK (*p* < 0.05).

### 3.3 Effects of dietary composite plant essential oils on small-intestinal morphology

**Figure 2:**
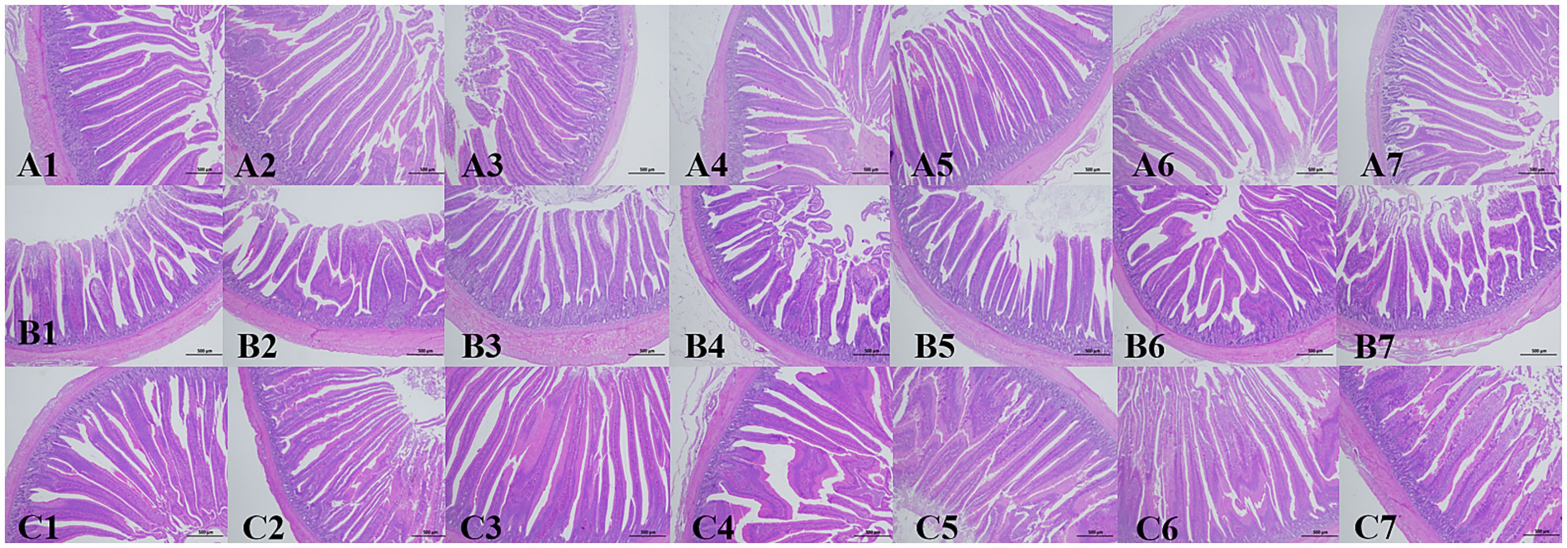
Histological morphology of the small intestine in AA broilers. Representative hematoxylin and eosin (H&E) stained sections of the intestine. Scale bar, 500 μm; magnification, 400×. **(A)** Jejunum; **(B)** ileum; **(C)** duodenum. Numbers 1–7 indicate dietary treatments: 1, CK (control, basal diet); 2, EO1 (EO 200 g/t); 3, EO2 (EO 600 g/t); 4, EO3 (EO 1200 g/t); 5, EO1+AG (EO 200 g/t + florfenicol 0.15 g/kg, days 7–21); 6, EO3+AG (EO 1200 g/t + florfenicol 0.15 g/kg, days 7–21); 7, AG (florfenicol 0.15 g/kg, days 7–21).

As shown in Supplementary Table 2 and Figure 2, ileal villus height (V) and villus to crypt ratio (V/C) did not differ significantly among EO1, EO2, EO3, EO1+AG, EO3+AG and AG versus CK (*p* > 0.05). Crypt depth (C) was increased in EO3+AG compared with CK (*p* < 0.05); other groups showed no significant change (*p* > 0.05). In the duodenum, villus height and crypt depth did not differ among groups (*p* > 0.05). The duodenal V/C ratio was higher in EO3+AG than in CK (*p* < 0.05), and was also higher in EO3+AG than in EO3, EO1+AG and AG (*p* < 0.05). In the jejunum, crypt depth and V/C ratio did not differ among treatments (*p* > 0.05). Jejunal villus height was greater in EO3+AG than in CK (*p* < 0.05).

### 3.4 Effects of dietary supplementation with a compound plant essential oil on the intestinal microbiota of broilers

#### 3.4.1 Microbial community composition in the cecum of 42-day-old AA broilers

##### 3.4.1.1 Phylum level relative abundance

The phylum level composition of cecal microbiota in 42-day-old AA broilers was examined (Supplementary Figure 2). Samples were grouped as A (CK), B (EO1), C (EO2), D (EO3), E (EO1+AG), F (EO3+AG) and G (AG). The relative abundances of the top ten phyla were plotted as bar charts. Across all groups, Firmicutes, Bacteroidetes, Proteobacteria, Tenericutes and Verrucomicrobia were dominant. Relative to the CK group, all treatments reduced the relative abundance of Firmicutes (EO1, *p* < 0.05; EO2 and EO3, *p* > 0.05; EO1+AG, EO3+AG, and AG, *p* < 0.05). The abundance of Bacteroidetes increased in EO1 (*p* < 0.05), EO3 (*p* < 0.05), EO1+AG (*p* < 0.05), EO3+AG (*p* < 0.05), and AG (*p* < 0.05), but decreased in EO2. Proteobacteria increased in EO2 (*p* < 0.05), EO1+AG, EO3+AG, and AG, but decreased in EO1 and EO3. Tenericutes decreased in EO2 and AG, whereas it increased in EO1, EO3, EO1+AG, and EO3+AG. Phyla with a relative abundance <1% included Verrucomicrobia, Cyanobacteria, Actinobacteria, Fusobacteria, and Acidobacteria.

##### 3.4.1.2 Family level relative abundance

**Figure 3:**
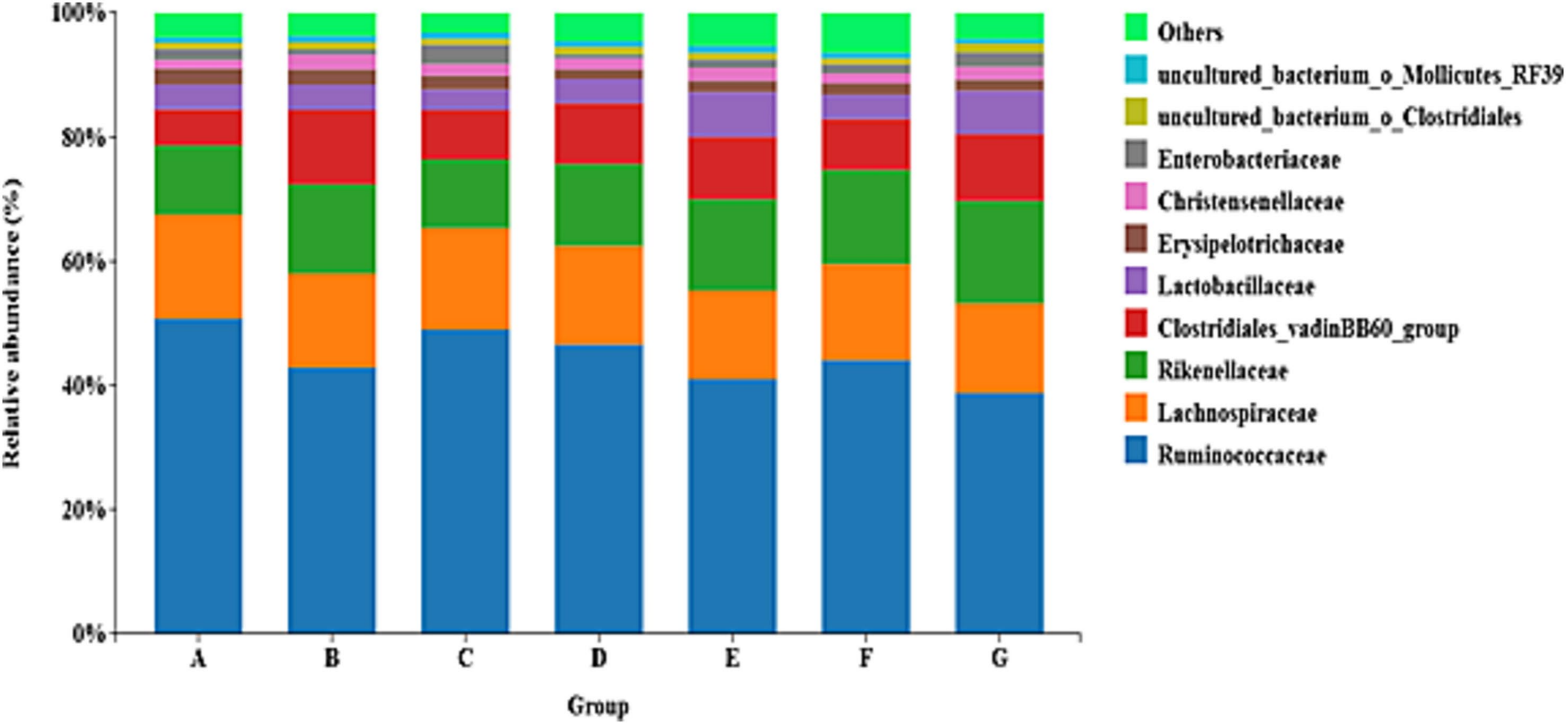
The family level abundance of cecal luminal microbiota. Treatments: (A) Control (CK), basal diet; (B) EO1, essential oils 200 g/t; (C) EO2, essential oils 600 g/t; (D) EO3, essential oils 1,200 g/t; (E) EO1+AG, EO 200 g/t + florfenicol (0.15 g/kg, days 7–21); (F) EO3+AG, EO 1200 g/t + florfenicol (0.15 g/kg, days 7–21); (G) AG, florfenicol (0.15 g/kg, days 7–21).

Family level composition of the cecal microbiota was assessed in 42-day-old AA broilers (Figure 3). Plot the relative abundances of the top 10 families of samples in different groups on the X-axis. The dominant families across all treatments were Ruminococcaceae, Lachnospiraceae, Rikenellaceae, the Clostridiales vadinBB60 group, Lactobacillaceae, Erysipelotrichaceae, Christensenellaceae and Enterobacteriaceae.

##### 3.4.1.3 Genus level relative abundance

Genus level cecal microbiota in 42-day-old AA broilers was examined (Supplementary Figure 3). Samples were grouped as A (CK), B (EO1), C (EO2), D (EO3), E (EO1+AG), F (EO3+AG) and G (AG). The ten most abundant genera across all groups were *Alistipes*, *Faecalibacterium, the Clostridiales vadinBB60 group, unclassified members of Lachnospiraceae, Ruminococcaceae UCG-014, Ruminococcus, Lactobacillus, the Ruminococcus torques group, Negativibacillus* and *the Eubacterium coprostanoligenes group.*

Relative to the CK group, *Alistipes* and *Clostridiales vadinBB60* were consistently enriched across all treatments, whereas *Faecalibacterium* was uniformly depleted (*p* < 0.05). *Unclassified Lachnospiraceae* declined in EO1, EO1+AG, EO3+AG and AG, but was unchanged or elevated in EO2 and EO3. *Ruminococcaceae UCG-014* increased under essential oil only treatments (EO1, EO2, EO3), but declined under the combined regimens (EO1+AG, *p* < 0.05; EO3+AG) and the antibiotic treatment (AG, *p* < 0.05). *Lactobacillus* showed treatment specific responses: it increased in EO1+AG (*p* < 0.05) and AG (*p* < 0.05), but decreased in EO2. N*egativibacillus* showed treatment dependent changes, being reduced in EO1, EO1+AG and EO3+AG while increasing in some EO treatments. These shifts indicated that the composite essential oil and florfenicol modulated key cecal genera, with distinct effects depending on dose and combination.

#### 3.4.2 Alpha diversity analysis of cecal microbiota

Alpha diversity metrics were calculated for each group. Coverage values approached 1.00 in all treatments, indicating that sequencing depth adequately reflected cecal microbial diversity. Rarefaction curves (Supplementary Figure 4) showed that observed OTU counts increased with sequencing effort and plateaued at high read depths, confirming that sequencing depth was sufficient.

**Figure 4:**
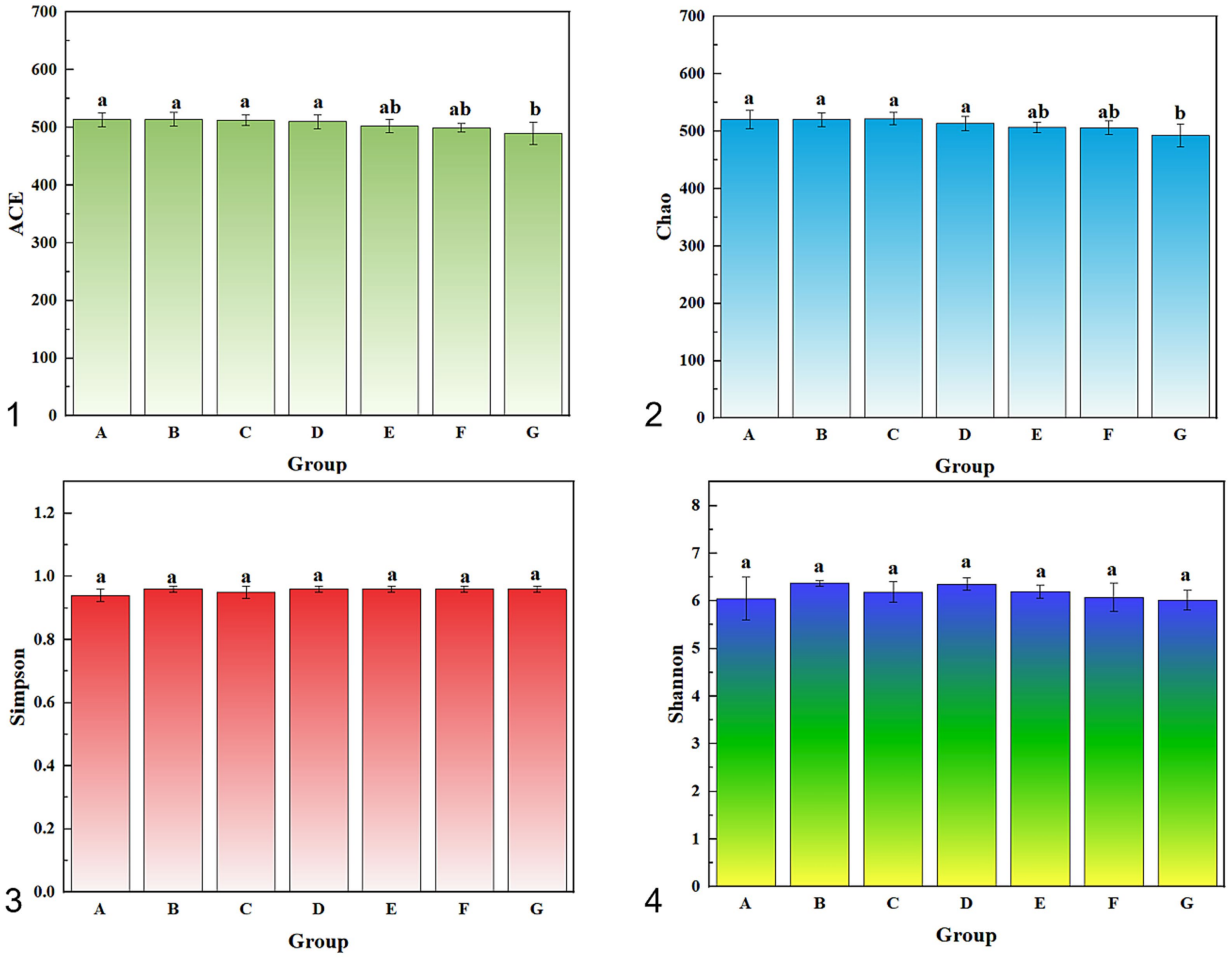
Alpha diversity index of sample. (1) Abundance-based Coverage Estimator; (2) Chao Index; (3) Simpson Diversity Index; (4) Shannon Diversity Index. Treatments: (A) Control (CK), basal diet; (B) EO1, essential oils 200 g/t; (C) EO2, essential oils 600 g/t; (D) EO3, essential oils 1,200 g/t; (E) EO1+AG, EO 200 g/t + florfenicol (0.15 g/kg, days 7–21); (F) EO3+AG, EO 1200 g/t + florfenicol (0.15 g/kg, days 7–21); (G) AG, florfenicol (0.15 g/kg, days 7–21). Different letters indicate significant differences among treatments (*p* < 0.05).

Statistical comparisons of diversity indices are summarized in Supplementary Table 3 and Figure 4. According to the ACE index, EO3+AG and AG differed significantly from CK (*p* < 0.05), and the essential oil only groups (EO1, EO2) differed from the combined antibiotic groups (EO3+AG, AG) (*p* < 0.05). Chao1 analysis showed that AG differed from CK and EO1 (*p* < 0.05), while EO2 differed from EO1+AG, EO3+AG and AG (*p* < 0.05). Shannon indices revealed significant differences between EO1 and both EO1+AG and AG (*p* < 0.05), and between EO3 and AG (*p* < 0.05). No significant differences were detected in Simpson indices among groups (*p* > 0.05).

## 4 Discussion

Numerous recent studies have explored the use of essential oils (EOs) in livestock and poultry production (10, 12, 42, 50–52). Accumulating evidence shows that EOs can promote growth by increasing average daily gain (ADG) and average daily feed intake (ADFI) (15). They also lower the feed to gain ratio (F/G) and reduce mortality (16). For example, dietary supplementation with 400 mg/kg EO improved broiler growth and reduced feed conversion (*p* < 0.05) (17). Zheng et al. reported that blends of monoglyceride lauric acid and cinnamaldehyde (350 and 500 mg/kg) increased ADG and improved F/G. These blends also enhanced intestinal morphology, improved antioxidant status and downregulated inflammatory markers; the 500 mg/kg dose produced the greatest benefit (18). Alagawany et al. found that lemongrass EO enhanced growth performance, lipid metabolism, immune responses and antioxidant capacity in quail, while reducing intestinal pathogens and overall health risk (19). Various plant EOs have been evaluated as dietary supplements for poultry, and their potential as eco-friendly alternatives to antibiotics in organic production has been reported (8, 20).

In the present trial, supplementation with a composite plant essential oil (carvacrol + thymol), either alone or combined with florfenicol, improved body weight in Arbor Acres broilers. From 22 to 42 d, average body weight (ABW) was higher in EO2, EO1+AG, EO3+AG and AG than in CK (*p* < 0.05). Average daily feed intake (ADFI) was lower in EO3+AG during this interval (*p* < 0.05). Over the entire 1–42 d period, ABW increased in EO2, EO1+AG, EO3+AG and AG (*p* < 0.05), while ADFI was higher in EO2 and EO3+AG (*p* < 0.05). No differences in average daily gain (ADG) were detected among groups (*p* > 0.05). These results indicate that EO2 and the combined EO + antibiotic regimens (EO1+AG, EO3+AG, AG) enhanced growth performance under the conditions tested, consistent with earlier reports (21–23). The effects may reflect the aroma and bioactivity of essential oils, which can increase feed palatability, stimulate salivation and promote gut motility, thereby supporting feed intake and physiological function. However, several studies reported no effect of EO supplementation on growth metrics (24–27). Such discrepancies likely arise from differences in EO composition, inclusion level, bird strain or experimental conditions.

Blood maintains internal homeostasis, and hematological indices reflect the interplay between nutrition and disease (28, 29). Total protein (TP) and albumin (ALB) are central to acid base balance, plasma oncotic pressure and tissue protein homeostasis. Aspartate aminotransferase (AST), a major hepatocellular transaminase, participates in amino acid catabolism and synthesis and serves as an indicator of amino acid metabolism (30, 31). Higher protein intake raises amino acid turnover and stimulates transaminase activity; elevated TP and ALB thus indicate a favorable nutritional state (8, 32). Glucose (GLU) reflects energy status and, when increased, is generally associated with improved immunity and stress resistance (33). In the present study, at 42 d, TP was significantly lower in EO1+AG and EO3+AG compared with CK (*p* < 0.05). ALB and globulin (GLB) were also reduced in EO1+AG and EO3+AG (*p* < 0.05). Serum cholesterol (CHOL) decreased in EO1+AG, EO3+AG and AG relative to CK (*p* < 0.05). These findings suggest that essential oils promote lipid catabolism. One plausible mechanism is that linoleic acid in thyme essential oil binds cholesterol and inhibits hepatic 3-hydroxy-3-methylglutaryl-coenzyme A (HMG-CoA) reductase. This inhibition may enhance conversion of cholesterol to bile acids and increase excretion, thereby lowering circulating lipids. Consistent with our results, Hong et al. reported that dietary supplementation with 125 ppm essential oil extract (blend of lemon, oregano and anise) and 100 ppm antibiotic (oxytetracycline) significantly reduced serum CHOL in broilers (*p* < 0.05). They also observed significant differences in very low density lipoprotein among control, essential oil and antibiotic groups, with the essential oil group showing the lowest values (14).

Intestinal structural integrity was evaluated by villus height (V), crypt depth (C) and the villus: crypt ratio (V/C) (8, 27, 34). Taller villi and shallower crypts indicate greater digestive and absorptive capacity. The intestine is both the main digestive organ and the largest immune organ; it is essential for nutrient digestion, absorption and defence against pathogens (35, 36). Modern broiler production exposes birds to multiple stressors for example, elevated ammonia, lighting regimes, stocking density, basal diet, transport, ambient temperature and humidity, noise and pathogens which readily induce intestinal damage, diarrhea and growth retardation (37–39). Dietary cinnamaldehyde increased jejunal villus height and tended to raise the jejunal V/C in heat stressed broilers, suggesting improved intestinal morphology and nutrient digestibility (40). Jiménez et al. reported that cinnamaldehyde increased villus height in piglets and protected villi from free radical damage via antioxidant effects (41). Similar benefits enhanced barrier integrity, increased villus height, reduced crypt depth and enlarged villus surface were reported after essential oil supplementation (42–44). In the present study, jejunal villus height and duodenal V/C were significantly increased in the EO3+AG group compared with CK (*p* < 0.05). These findings indicate that the tested essential oil preparation, at the studied dose, can support intestinal development in AA broilers.

The gut microbiota of livestock and poultry normally exists in a dynamic equilibrium and has a strong self-repair capacity. Disturbances such as environmental stress, dietary shifts, improper antibiotic use and pathogen overgrowth can damage the mucosa and trigger disease. Dietary inclusion of EOs has been reported to reshape microbial composition, improve nutrient digestibility and support immune function. EOs are proposed to suppress harmful taxa (e.g., *Clostridium perfringens*, *Escherichia coli*) while promoting beneficial genera (e.g., *Lactobacillus*, *Bifidobacterium*), thus optimizing the microbial environment and preserving mucosal integrity. The phylum Proteobacteria includes many opportunistic pathogens, such as *E. coli* and *Salmonella*, which can impair productivity (17, 42, 45). By contrast, *Lactobacillus* is regarded as beneficial in broiler intestines because it acidifies the lumen, inhibits pathogens and contributes to gut health. Consistent with these observations, Irawan et al. found that a carvacrol–thymol blend reduced pathogenic *E. coli* in the cecum and ileum and modulated *Lactobacillus* populations in broilers (32).

The taxonomic composition of the gut microbiota is shaped by several factors, including host age, diet, anatomical site and antibiotic exposure (46, 47). Many studies report that Bacteroidetes, Firmicutes and Proteobacteria are the dominant phyla, with Bacteroidetes and Firmicutes typically being the most abundant (17, 48, 49). These groups play central roles in nutrient absorption and energy metabolism and collectively support host nutrient uptake and energy storage. In broilers, the relative abundances of Verrucomicrobia, Cyanobacteria, Actinobacteria, Fusobacteria and Acidobacteria did not decline markedly, suggesting a stable community structure. Changes in host health therefore often reflect shifts in specific pathogenic taxa, rather than broad reductions in overall microbial diversity.

## 5 Conclusion

Supplementation of the basal diet with the composite essential oil (carvacrol + thymol) at 600 g/t, or a regimen of florfenicol (0.15 g/kg during the starter phase) followed by 1,200 g/t essential oil in the grower phase, significantly increased average body weight (ABW) and average daily feed intake (ADFI). Average daily gain (ADG) and feed: gain ratio (F/G) were not affected. The combined regimen of early antibiotic use followed by later essential oil supplementation (200 g/t or 1,200 g/t) significantly reduced total protein (TP), albumin (ALB), globulin (GLB) and total cholesterol (CHOL). Histological analysis showed that 1,200 g/t essential oil supplementation increased jejunal villus height and duodenal villus: crypt ratio (V/C). Cecal 16S rRNA sequencing revealed an increased relative abundance of Bacteroidetes and a concomitant decrease in Firmicutes, indicating a shift in microbial composition. Taken together, the composite essential oil demonstrated potential as an antibiotic alternative to improve production performance, support intestinal development and modulate the gut microbiota under the tested dosing schedules. Further systematic studies on dose response relationships, long term safety, component interactions (synergy/antagonism) and delivery strategies are warranted to enable reproducible and scalable application in antibiotic free poultry production.

## Data Availability

The datasets presented in this study can be found in online repositories. The names of the repository/repositories and accession number(s) can be found in the article/Supplementary material.

## References

[1] Fotou E Moulasioti V Papadopoulos AG Kyriakou D Boti M-E Moussis V . Effect of farming system type on broilers’ antioxidant status, performance, and carcass traits: an industrial-scale production study. Sustainability. (2024) 16:4782. doi: 10.3390/SU16114782

[2] Nduku X Stempa T Lungu SN Ndobeni TN Mlambo V. Prospects for antibiotic-free poultry production in South Africa: an analysis of the enablers and stumbling blocks. One Health. (2025) 21:21101144. doi: 10.1016/J.ONEHLT.2025.101144 PMC12305726 40735741

[3] Selim S Megeid ASN Alhotan AR Ebrahim A Hussein E. Nutraceuticals vs. antibiotic growth promoters: differential impacts on performance, meat quality, blood lipids, cecal microbiota, and organ histomorphology of broiler chicken. Poult Sci. (2024) 103:103971. doi: 10.1016/J.PSJ.2024.103971 38941788 PMC11260365

[4] Sousa M Machado I Simões CL Simões M. Biocides as drivers of antibiotic resistance: a critical review of environmental implications and public health risks. Environ Sci Ecotechnol. (2025) 25:25100557. doi: 10.1016/J.ESE.2025.100557 PMC11995807 40230384

[5] Carvalho FA de Moraes NV Crotti AEM Crevelin EJ Santos AGD. *Casearia* essential oil: an updated review on the chemistry and pharmacological activities. Chem Biodivers. (2023) 20:e202300492. doi: 10.1002/CBDV.202300492 37410861

[6] Vlaicu AP Untea EA Panaite DT Vlaicu P Untea A Panaite T . Effect of basil, thyme and sage essential oils as phytogenic feed additives on production performances, meat quality and intestinal microbiota in broiler chickens. Agriculture. (2023) 13:874. doi: 10.3390/AGRICULTURE13040874

[7] Wińska K Mączka W Łyczko J Grabarczyk M Czubaszek A Szumny A. Essential oils as antimicrobial agents—myth or real alternative? Molecules. (2019) 24:2130–11. doi: 10.3390/molecules24112130, 31195752 PMC6612361

[8] Abd El-Hack ME El-Saadony MT Saad AM Salem HM Ashry NM Abo Ghanima MM . Essential oils and their nanoemulsions as green alternatives to antibiotics in poultry nutrition: a comprehensive review. Poult Sci. (2022) 101:101584. doi: 10.1016/J.PSJ.2021.101584, 34942519 PMC8695362

[9] Hassan AHA Youssef IMI Atty ASN Abdel-Daim ASA. Effect of thyme, ginger, and their nano-particles on growth performance, carcass characteristics, meat quality and intestinal bacteriology of broiler chickens. BMC Vet Res. (2024) 20:269–9. doi: 10.1186/S12917-024-04101-Z 38907235 PMC11193295

[10] Majid G Asiye A Shahrzad A Basiratpour A Zokaei M Derakhshan M. Thymol and carvacrol supplementation in poultry health and performance. Vet Med Sci. (2021) 8:267–88. doi: 10.1002/VMS3.663 34761555 PMC8788968

[11] Chavira SJ García BBH. Essential oils, chemical compounds, and their effects on the gut microorganisms and broiler chicken production: review. Agriculture. (2024) 14:1864-1864. doi: 10.3390/AGRICULTURE14111864

[12] Li XL Xiao CC Ke YZ Tian G Ding X Bai S . Effects of thymol and carvacrol eutectic on growth performance, serum biochemical parameters, and intestinal health in broiler chickens. Animal. (2023) 13:2242. doi: 10.3390/ANI13132242 PMC10340057 37444040

[13] Yang J Li C Jiang S Fu Y Zhou G Gao Y . Dietary thymol–carvacrol cocrystal supplementation improves growth performance, antioxidant status, and intestinal health in broiler chickens. Antioxidants. (2025) 14:1323–3. doi: 10.3390/ANTIOX14111323, 41300480 PMC12649561

[14] Hong JC Steiner T Aufy A Lien T-F. Effects of supplemental essential oil on growth performance, lipid metabolites and immunity, intestinal characteristics, microbiota and carcass traits in broilers. Livest Sci. (2012) 144:253–62. doi: 10.1016/j.livsci.2011.12.008

[15] He CQ Liu Y Dai Y He C Yao Y Li M . The influence of plant essential oil/palygorskite composite on growth performance, blood parameters and intestinal morphology of broiler chickens. Ital J Anim Sci. (2023) 22:1073–82. doi: 10.1080/1828051X.2023.2271921

[16] Reis JH Gebert RR Barreta M Baldissera MD dos Santos ID Wagner R . Effects of phytogenic feed additive based on thymol, carvacrol and cinnamic aldehyde on body weight, blood parameters and environmental bacteria in broilers chickens. Microb Pathog. (2018) 125:168–76. doi: 10.1016/j.micpath.2018.09.015, 30205193

[17] Ding Y Hu Y Yao X He Y Chen J Wu J . Dietary essential oils improves the growth performance, antioxidant properties and intestinal permeability by inhibiting bacterial proliferation, and altering the gut microbiota of yellow-feather broilers. Poult Sci. (2022) 101:102087–7. doi: 10.1016/J.PSJ.2022.102087, 36095866 PMC9472070

[18] Zheng CJ Xiao GS Yan X Zheng C Xiao G Qiu T . Complex of lauric acid monoglyceride and cinnamaldehyde ameliorated subclinical necrotic enteritis in yellow-feathered broilers by regulating gut morphology, barrier, inflammation and serum biochemistry. Animals. (2023) 13:516-516. doi: 10.3390/ANI13030516, 36766404 PMC9913842

[19] Alagawany M El-Saadony MT Elnesr SS Farahat M Attia G Madkour M . Use of lemongrass essential oil as a feed additive in quail's nutrition: its effect on growth, carcass, blood biochemistry, antioxidant and immunological indices, digestive enzymes and intestinal microbiota. Poult Sci. (2021) 100:101172-101172. doi: 10.1016/J.PSJ.2021.101172, 33951594 PMC8111249

[20] Chowdhury S Mandal PG Patra KA Kumar P Samanta I Pradhan S . Different essential oils in diets of broiler chickens: 2. Gut microbes and morphology, immune response, and some blood profile and antioxidant enzymes. Anim Feed Sci Technol. (2018) 236:39–47. doi: 10.1016/j.anifeedsci.2017.12.003

[21] Agung I Cecep H Anuraga J Ratriyanto A. Essential oils as growth-promoting additives on performance, nutrient digestibility, cecal microbes, and serum metabolites of broiler chickens: a meta-analysis. Asian Australas J Anim Sci. (2020) 34:1499–513. doi: 10.5713/AB.20.0668 PMC8495342 33332937

[22] Chen Y Wang J Yu L Xu T Zhu N. Microbiota and metabolome responses in the cecum and serum of broiler chickens fed with plant essential oils or virginiamycin. Sci Rep. (2020) 10:5382. doi: 10.1038/s41598-020-60135-x, 32214106 PMC7096418

[23] Gholami-Ahangaran M Ahmadi-Dastgerdi A Karimi-Dehkordi M. Thymol and carvacrol; as antibiotic alternative in green healthy poultry production. Plant Biotechnol Persa. (2020) 2:22–5. doi: 10.29252/pbp.2.1.22

[24] Abouelezz K Abou-Hadied M Yuan J Elokil AA Wang G Wang S . Nutritional impacts of dietary oregano and Enviva essential oils on the performance, gut microbiota and blood biochemicals of growing ducks. Animal. (2019) 13:2216–22. doi: 10.1017/S1751731119000508, 30914073

[25] El-Hack M Alagawany M. Performance, egg quality, blood profile, immune function, and antioxidant enzyme activities in laying hens fed diets with thyme powder. J Anim Feed Sci. (2015) 24:127–33. doi: 10.22358/jafs/65638/2015

[26] Fernandez EM Kembro MJ Ballesteros LM Caliva JM Marin RH Labaque MC . Dynamics of thymol dietary supplementation in quail (*Coturnix japonica*): linking bioavailability, effects on egg yolk total fatty acids and performance traits. PLoS One. (2019) 14:e0216623. doi: 10.1371/journal.pone.0216623 31071185 PMC6508865

[27] Ruan D Fan Q Fouad MA Sun Y Huang S Wu A . Effects of dietary oregano essential oil supplementation on growth performance, intestinal antioxidative capacity, immunity, and intestinal microbiota in yellow-feathered chickens. J Anim Sci. (2021) 99:1–11. doi: 10.1093/JAS/SKAB033 PMC7918158 33544855

[28] Akrami R Gharaei A Mansour RM Galeshi A. Effects of dietary onion (*Allium cepa*) powder on growth, innate immune response and hemato–biochemical parameters of beluga (*Huso huso* Linnaeus, 1754) juvenile. Fish Shellfish Immunol. (2015) 45:828–34. doi: 10.1016/j.fsi.2015.06.005 26067169

[29] Csilla T Edina S Bohumil B Nagy O. Changes of total protein and protein fractions in broiler chickens during the fattening period. Vet World. (2019) 12:598–604. doi: 10.14202/vetworld.2019.598-604 31190717 PMC6515826

[30] Kaiser JC Reider H Pabilonia KL Moore AR. Establishment of biochemical reference values for backyard chickens in Colorado (Gallus gallus domesticus). Vet Clin Pathol. (2022) 51:577–84. doi: 10.1111/vcp.13136 35488187 PMC10084313

[31] Qaid MM Al Garadi MA. Protein and amino acid metabolism in poultry during and after heat stress: A review. Animals. (2021) 11:1167–7. doi: 10.3390/ANI11041167, 33921616 PMC8074156

[32] Irawan A Hidayat C Jayanegara A Ratriyanto A. Essential oils as growth-promoting additives on performance, nutrient digestibility, cecal microbes, and serum metabolites of broiler chickens: a meta-analysis. Asian Australas J Anim Sci. (2020) 34:1499–513. doi: 10.5713/AB.20.0668 PMC8495342 33332937

[33] Ariyo WO Kwakye J Sovi S Aryal B Ghareeb AFA Hartono E . Glucose supplementation improves performance and alters glucose transporters’ expression in *pectoralis major*of heat-stressed chickens. Animals. (2023) 13:2911. doi: 10.3390/ANI13182911 37760311 PMC10525872

[34] Chamorro S Romero C Brenes A Sánchez-Patán F Bartolomé B Viveros A . Impact of a sustained consumption of grape extract on digestion, gut microbial metabolism and intestinal barrier in broiler chickens. Food Funct. (2019) 10:1444–54. doi: 10.1039/c8fo02465k, 30768097

[35] Balasubramanian B Liu CW. Editorial: gut microbiota: allied with livestock nutrition, health, and welfare. Front Vet Sci. (2024):111413671:11. doi: 10.3389/FVETS.2024.1413671 PMC11130472 38807938

[36] Franciosini MP Casagrande-proietti P Forte C Beghelli D Acuti G Zanichelli D . Effects of oregano (*Origanum vulgare* L.) and rosemary (*Rosmarinus officinalis* L.) aqueous extracts on broiler performance, immune function and intestinal microbial population. J Appl Anim Res. (2016) 44:474–9. doi: 10.1080/09712119.2015.1091322

[37] Goel A Ncho CM Choi YH. Regulation of gene expression in chickens by heat stress. J. Anim. Sci. Biotechnol. (2021) 12:11. doi: 10.1186/s40104-020-00523-5, 33431031 PMC7798204

[38] Tian H Guo Y Ding M Su A Li W Tian Y . Identification of genes related to stress affecting thymus immune function in a chicken stress model using transcriptome analysis. Res Vet Sci. (2021) 138:90–9. doi: 10.1016/J.RVSC.2021.06.006, 34126450

[39] Wasti S Sah N Mishra B. Impact of heat stress on poultry health and performances, and potential mitigation strategies. Animals. (2020) 10:1266. doi: 10.3390/ani10081266, 32722335 PMC7460371

[40] Kang H Wang Q Yu H Guo Q Weber LI Wu W . Validating the use of a newly developed cinnamaldehyde product in commercial broiler production. Poult Sci. (2024) 103:103625–5. doi: 10.1016/J.PSJ.2024.103625, 38507831 PMC10966097

[41] Jiménez MJ Roger B Sabine S Bracarense APFRL. Ingestion of organic acids and cinnamaldehyde improves tissue homeostasis of piglets exposed to enterotoxic *Escherichia coli* (ETEC). J Anim Sci. (2020) 98:skaa012. doi: 10.1093/jas/skaa012 31943046 PMC7190117

[42] Li Z Jin X Wu Q Long L Li Y Zhang Q . Effects of encapsulated thymol and carvacrol mixture on growth performance, antioxidant capacity, immune function and intestinal health of broilers. Ital J Anim Sci. (2022) 21:1651–9. doi: 10.1080/1828051X.2022.2151944

[43] Zhang K Yan F Keen C Waldroup PW. Evaluation of microencapsulated essential oils and organic acids in diets for broiler chickens. Int J Poult Sci. (2005) 4:612. doi: 10.3923/ijps.2005.612.619

[44] Zou Y Xiang Q Wang J Peng J Wei H. Oregano essential oil improves intestinal morphology and expression of tight junction proteins associated with modulation of selected intestinal bacteria and immune status in a pig model. Biomed Res Int. (2016) 2016:5436738. doi: 10.1155/2016/5436738, 27314026 PMC4903144

[45] Pan JF Zhu YL Abdel-Samie MA Pan J Zhu Y Li C . Biological properties of essential oil emphasized on the feasibility as antibiotic substitute in feedstuff. Grain Oil Sci. Technol. (2023) 6:10–23. doi: 10.1016/J.GAOST.2022.11.001

[46] Sun B Hou LY Yang Y. The development of the gut microbiota and short-chain fatty acids of layer chickens in different growth periods. Front Vet Sci. (2021) 8:8666535–5. doi: 10.3389/FVETS.2021.666535, 34277754 PMC8284478

[47] Wang X Tsai T Deng F Wei X Chai J Knapp J . Longitudinal investigation of the swine gut microbiome from birth to market reveals stage and growth performance associated bacteria. Microbiome. (2019) 7:109. doi: 10.1186/s40168-019-0721-7, 31362781 PMC6664762

[48] Stanley D Geier SM Denman ES Haring VR Crowley TM Hughes RJ . Identification of chicken intestinal microbiota correlated with the efficiency of energy extraction from feed. Vet Microbiol. (2013) 164:85–92. doi: 10.1016/j.vetmic.2013.01.030 23434185

[49] Zhao CZ Hu JL Li Q Zhao C Hu J Fang Y . Transfer of nitrogen and phosphorus from cattle manure to soil and oats under simulative cattle manure deposition. Front Microbiol. (2022) 13:916610-916610. doi: 10.3389/FMICB.2022.916610 35774448 PMC9238326

[50] Bao H Xue Y Zhang Y Tu F Wang R Cao Y . Encapsulated essential oils improve the growth performance of meat ducks by enhancing intestinal morphology, barrier function, antioxidant capacity and the cecal microbiota. Antioxidants. (2023) 12:253–3. doi: 10.3390/ANTIOX12020253, 36829812 PMC9952412

[51] Lian HZ Fei G Jun WG Li H Xia F Bai H . Potential of aromatic plant-derived essential oils for the control of foodborne bacteria and antibiotic resistance in animal production: a review. Antibiotics. (2022) 11:1673–3. doi: 10.3390/ANTIBIOTICS11111673 36421318 PMC9686951

[52] Meligy AMA Marwa AE-H I Yonis AE Elhaddad GY Abdel-Raheem SM El-Ghareeb WR . Liposomal encapsulated oregano, cinnamon, and clove oils enhanced the performance, bacterial metabolites antioxidant potential, and intestinal microbiota of broiler chickens. Poult Sci. (2023) 102:102683–3. doi: 10.1016/J.PSJ.2023.102683 37120892 PMC10173274

