# Supplementary material for: Effects of dietary supplementation with a thymol-carvacrol blend on growth performance and intestinal health of poultry
Source: Front Vet Sci. 2026 Jan 12;12:1739666. doi: 10.3389/fvets.2025.1739666 (PMC12832459; doi:10.3389/fvets.2025.1739666)
Supplement: Supplementary file 1 [file Data_Sheet_1.docx]

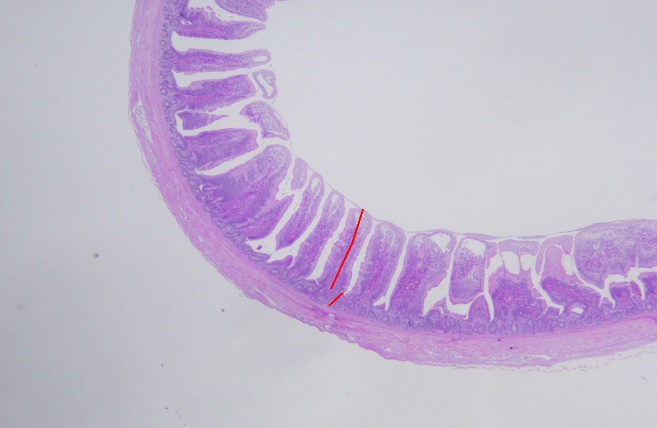


**Note:** Representative hematoxylin and eosin (H&E)-stained intestinal sections used for the measurement of villus height and crypt depth. Scale bar, 500 μm; magnification, 400×. The red arrow indicates villus height, and the yellow arrow indicates crypt depth.

**Figure S1** Analysis of villus height and crypt depth at 42 days old

**
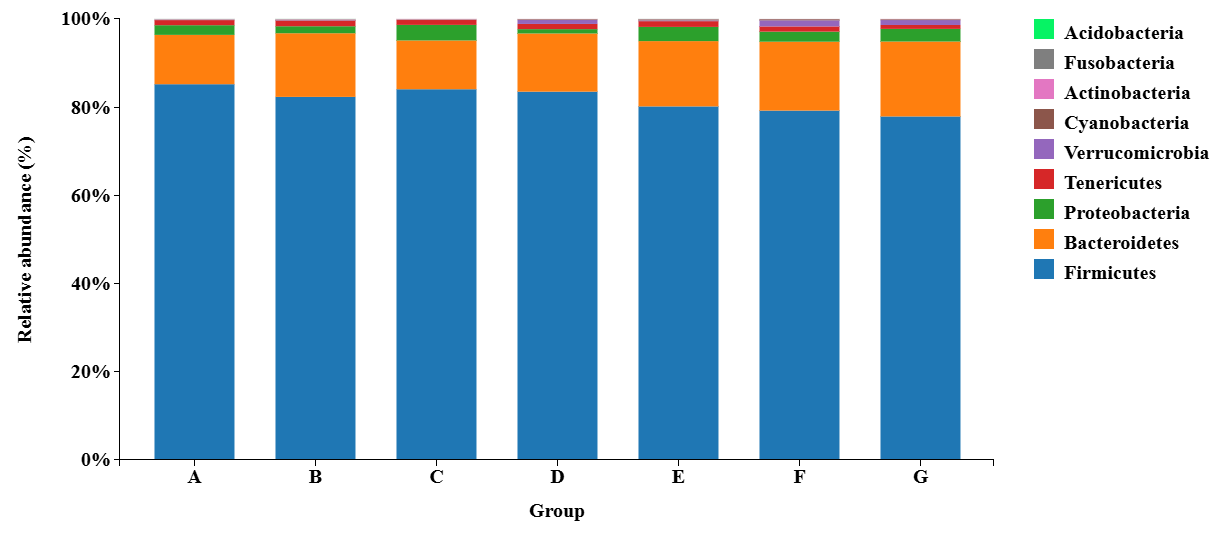
**

Note: (A) Control (CK), basal diet; (B) EO1, essential oils 200 g/t; (C) EO2, essential oils 600 g/t; (D) EO3, essential oils 1200 g/t; (E) EO1+AG, EO 200 g/t + florfenicol (0.15 g/kg, days 7–21); (F) EO3+AG, EO 1200 g/t + florfenicol (0.15 g/kg, days 7–21); (G) AG, florfenicol (0.15 g/kg, days 7–21).

**Figure S2** Phylum level abundance of cecal luminal microbiota

**
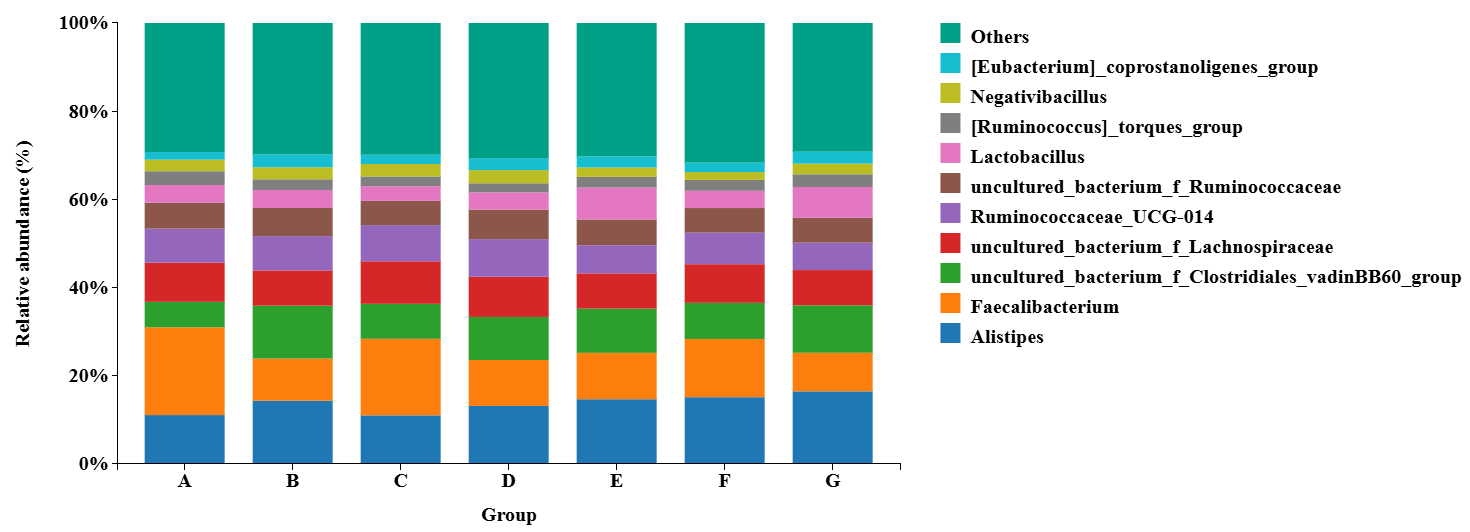
**

Note: (A) Control (CK), basal diet; (B) EO1, essential oils 200 g/t; (C) EO2, essential oils 600 g/t; (D) EO3, essential oils 1200 g/t; (E) EO1+AG, EO 200 g/t + florfenicol (0.15 g/kg, days 7–21); (F) EO3+AG, EO 1200 g/t + florfenicol (0.15 g/kg, days 7–21); (G) AG, florfenicol (0.15 g/kg, days 7–21).


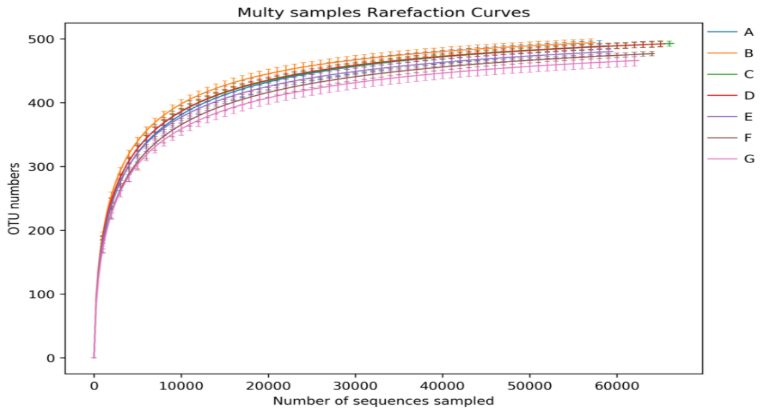
**Figure S3** Genus level abundance of cecal luminal microbiota

Note: (A) Control (CK), basal diet; (B) EO1, essential oils 200 g/t; (C) EO2, essential oils 600 g/t; (D) EO3, essential oils 1200 g/t; (E) EO1+AG, EO 200 g/t + florfenicol (0.15 g/kg, days 7–21); (F) EO3+AG, EO 1200 g/t + florfenicol (0.15 g/kg, days 7–21); (G) AG, florfenicol (0.15 g/kg, days 7–21).

**Figure S4** Rarefaction Curve of sample
