# Supplementary material for: Effects of dietary supplementation with a thymol-carvacrol blend on growth performance and intestinal health of poultry
Source: Front Vet Sci. 2026 Jan 12;12:1739666. doi: 10.3389/fvets.2025.1739666 (PMC12832459; doi:10.3389/fvets.2025.1739666)
Supplement: Supplementary file 2 [file Table_1.docx]

**Table S1** Effects of Dietary compound Plant essential Oils on Serum biochemical Indexes of AA broilers

|  | Items | A（CK） | B（EO1） | C（EO2） | D（EO3） | E（EO1+AG） | F（EO3+AG） | G（AG） |
| --- | --- | --- | --- | --- | --- | --- | --- | --- |
| 21  days | TP | 2.60±0.12 ^a^ | 2.74±0.05 ^a^ | 2.76±0.08 ^a^ | 2.78±0.11 ^a^ | 2.81±0.14 ^a^ | 2.52±0.16 ^a^ | 2.52±0.19 ^a^ |
|  | ALB | 1.20±0.05 ^b^ | 1.28±0.04 ^ab^ | 1.26±0.06 ^ab^ | 1.35±0.08 ^ab^ | 1.39±0.06 ^a^ | 1.31±0.07 ^ab^ | 1.31±0.08 ^ab^ |
|  | GLB | 1.40±0.1 ^a^ | 1.46±0.17 ^a^ | 1.49±0.16 ^a^ | 1.42±0.15 ^a^ | 1.41±0.11 ^a^ | 1.21±0.12^a^ | 1.20±0.16 ^a^ |
|  | GLU | 159.21±14.86 ^ab^ | 129.2±15.34 ^b^ | 153.54±13.22 ^ab^ | 156.92±14.12 ^ab^ | 196.58±14.65 ^a^ | 185.41±12.65 ^a^ | 195.15±13.98 ^a^ |
|  | AST | 272.05±32.99 ^ab^ | 253.50±34.90 ^b^ | 333.11±28.38 ^a^ | 312.93±32.67 ^ab^ | 241.18±31.49 ^b^ | 249.46±27.95 ^b^ | 237.19±25.12 ^b^ |
|  | GGT | 14.73±2.76 ^ab^ | 16.37±2.11 ^ab^ | 13.85±3.40 ^ab^ | 17.93±1.3 ^a^ | 15.12±1.63 ^ab^ | 12.97±1.55 ^b^ | 12.72±1.72 ^b^ |
|  | CHOL | 119.87±6.48 ^b^ | 122.85±8.21 ^ab^ | 117.71±7.11 ^b^ | 128.06±5.29 ^ab^ | 138.97±8.74 ^a^ | 133.56±8.32 ^ab^ | 131.17±7.98 ^ab^ |
|  | TG | 29.20±4.48 ^ab^ | 28.58±4.37 ^ab^ | 23.78±7.22 ^b^ | 31.61±3.78 ^ab^ | 37.10±3.22 ^a^ | 29.33±5.81 ^ab^ | 36.23±6.79 ^ab^ |
|  | IL－6 | 4.11±0.44 ^a^ | 3.55±0.41^a^ | 2.22±0.64^bc^ | 2.71±0.32^b^ | 2.60 ±0.70 ^b^ | 1.68 ±0.52 ^cd^ | 1.29±0.29 ^d^ |
|  | IL－10 | 1.95±0.32 ^a^ | 1.93 ±0.42 ^a^ | 1.77±0.37 ^a^ | 2.14±0.31^a^ | 3.17 ±0.22 ^b^ | 2.17 ±0.29 ^a^ | 2.00±0.26 ^a^ |
| 42  days | TP | 3.10±0.14^a^ | 3.18±0.15 ^a^ | 3.30±0.12 ^a^ | 2.91±0.26 ^ab^ | 2.41±0.24 ^b^ | 2.32±0.15 ^b^ | 2.72±0.24 ^ab^ |
|  | ALB | 1.22±0.14 ^ab^ | 1.22±0.14 ^ab^ | 1.23±0.13 ^a^ | 1.15±0.12 ^abc^ | 0.95±0.06 ^d^ | 0.99±0.10 ^cd^ | 1.03±0.13 ^bcd^ |
|  | GLB | 1.88±0.14 ^abc^ | 1.96±0.17 ^ab^ | 2.07±0.14 ^a^ | 1.76±0.21 ^abc^ | 1.46±0.17 ^bc^ | 1.33±0.09 ^c^ | 1.69±0.24 ^abc^ |
|  | GLU | 140.55±16.25 ^a^ | 158.99±13.22 ^a^ | 135.24±20.83 ^a^ | 173.36±18.62 ^a^ | 146.80±13.29 ^a^ | 134.92±21.79 ^a^ | 152.55±13.24 ^a^ |
|  | AST | 405.48±30.21 ^a^ | 450.12±62.91 ^a^ | 420.62±39.62 ^a^ | 398.93±36.88 ^a^ | 370.08±31.83 ^a^ | 340.70±50.79 ^a^ | 326.65±63.78 ^a^ |
|  | GGT | 7.89±2.27 ^b^ | 10.83±2.86 ^ab^ | 9.42±3.11 ^ab^ | 12.76±2.96 ^ab^ | 12.64±1.96 ^ab^ | 12.08±2.15 ^ab^ | 15.07±3.22 ^a^ |
|  | CHOL | 108.12±5.91 ^ab^ | 108.69±5.33 ^ab^ | 118.67±6.77 ^a^ | 97.65±9.21 ^bc^ | 83.76±7.84 ^c^ | 85.82±6.26 ^c^ | 84.83±8.04 ^c^ |
|  | TG | 28.57±4.77 ^ab^ | 30.80±5.21 ^ab^ | 31.63±5.55 ^ab^ | 32.87±4.82 ^a^ | 27.05±5.82 ^ab^ | 21.18±4.22 ^b^ | 28.95±3.92 ^ab^ |
|  | IL－6 | 5.70±2.35^a^ | 4.76 ±1.41 ^a^ | 11.66 ±3.22 ^a^ | 19.58±3.12^b^ | 24.41 ±2.98 ^b^ | 8.12±3.22^a^ | 15.44±3.68^ab^ |
|  | IL－10 | 12.82±0.66 | 10.95±0.63 | 6.76±0.74 | 12.52±0.81 | 10.24±0.69 | 10.22±0.72 | 7.45±0.7 |

Note: Values within a row sharing the same or no lowercase superscript letters are not significantly different (P > 0.05), whereas different lowercase letters indicate significant differences (P < 0.05). Abbreviations: TP, total protein; ALB, albumin; GLB, globulin; GLU, glucose; AST, aspartate aminotransferase; GGT, γ-glutamyl transferase; CHOL, total cholesterol; TG, triglycerides; IL-6, interleukin-6; IL-10, interleukin-10. Treatments: (A) Control (CK), basal diet; (B) EO1, essential oils 200 g/t; (C) EO2, essential oils 600 g/t; (D) EO3, essential oils 1200 g/t; (E) EO1+AG, EO 200 g/t + florfenicol (0.15 g/kg, days 7-21); (F) EO3+AG, EO 1200 g/t + florfenicol (0.15 g/kg, days 7-21); (G) AG, florfenicol (0.15 g/kg, days 7-21).

**Table S2** Effects of dietary compound plant essential oils on the morphology of duodenum, jejunum and ileum in AA broilers (n=8)

| Items |  |  |  | Groups |  |  |  |
| --- | --- | --- | --- | --- | --- | --- | --- |
|  | A（CK） | B（EO1） | C（EO2） | D（EO3） | E（EO1+AG） | F（EO3+AG） | G（AG） |
| Ileum |  |  |  |  |  |  |  |
| Villus height( V/mm) | 1.12±0.23 | 1.07±0.27 | 1.06±0.22 | 1.07±0.24 | 1.18±0.28 | 1.09±0.15 | 1.02±0.20 |
| Crypt depth( C/mm) | 0.18±0.03^ab^ | 0.17±0.01^ab^ | 0.16±0.02^a^ | 0.18±0.03^ab^ | 0.18±0.02^ab^ | 0.19±0.03^b^ | 0.17±0.03^ab^ |
| V/C | 6.22±1.08 | 6.36±1.33 | 6.71±1.39 | 6.05±1.26 | 6.56±0.95 | 5.79±0.54 | 5.99±0.71 |
| Duodenum |  |  |  |  |  |  |  |
| Villus height( V/mm) | 1.63±0.31 | 1.73±0.04 | 1.97 ±0.29 | 1.85±0.45 | 1.89±0.16 | 1.98±0.28 | 1.88±0.21 |
| Crypt depth( C/mm) | 0.19±0.01 | 0.17±0.03 | 0.19±0.02 | 0.18±0.02 | 0.19±0.02 | 0.16±0.03 | 0.18±0.01 |
| V/C | 8.62±1.48^a^ | 10.18±1.54^ab^ | 10.36±1.56^ab^ | 10.17±1.96^ab^ | 10.07±1.21^ab^ | 12.71±2.55^b^ | 10.30±0.78^a^ |
| Jejunum |  |  |  |  |  |  |  |
| Villus height( V/mm) | 1.64±0.28^ab^ | 1.70±0.43^ab^ | 1.55±0.14^ab^ | 1.69±0.21^ab^ | 1.46±0.35^a^ | 1.94±0.23^b^ | 1.67±0.34^ab^ |
| Crypt depth( C/mm) | 0.20±0.01 | 0.19±0.02 | 0.19±0.02 | 0.17±0.02 | 0.20±0.04 | 0.20±0.02 | 0.20±0.03 |
| V/C | 8.34±1.33 | 9.17±2.89 | 8.18±0.79 | 9.79±2.25 | 7.42±2.16 | 9.56±1.56 | 8.39±1.78 |

Note: In the same row, values with the same or no letter superscripts indicate no significant difference (P > 0.05), while those with different lowercase letter superscripts denote a significant difference (P < 0.05).

(A) Control (CK), basal diet; (B) EO1, essential oils 200 g/t; (C) EO2, essential oils 600 g/t; (D) EO3, essential oils 1200 g/t; (E) EO1+AG, EO 200 g/t + florfenicol (0.15 g/kg, days 7–21); (F) EO3+AG, EO 1200 g/t + florfenicol (0.15 g/kg, days 7–21); (G) AG, florfenicol (0.15 g/kg, days 7–21).

**Table S3** Alpha diversity index of each sample

| Diversity index | A（CK） | B（EO1） | C（EO2） | D（EO3） | E（EO1+AG） | F（EO3+AG） | G（AG） |
| --- | --- | --- | --- | --- | --- | --- | --- |
| 42 days |  |  |  |  |  |  |  |
| ACE | 513.48±11.93 ^a^ | 514.24±11.51 ^a^ | 512.72±9.42 ^a^ | 510.01±12.04 ^a^ | 502.04±11.25 ^ab^ | 499.16±7.15 ^ab^ | 489.74±19.73 ^b^ |
| Chao | 520.91±15.77 ^a^ | 520.30±12.12 ^a^ | 522.20±10.58 ^a^ | 514.27±12.34 ^a^ | 506.75±9.12 ^ab^ | 505.99±11.83 ^ab^ | 492.72±19.84 ^b^ |
| Simpson | 0.94±0.02 | 0.96±0.00 | 0.95±0.02 | 0.96±0.01 | 0.96±0.01 | 0.96±0.01 | 0.96±0.01 |
| Shannon | 6.05±0.45 | 6.37±0.06 | 6.19±0.22 | 6.35±0.13 | 6.20±0.14 | 6.08±0.30 | 6.02±0.21 |

Note: In the same row, values with the same or no letter superscripts indicate no significant difference (P > 0.05), while those with different lowercase letter superscripts denote a significant difference (P < 0.05).

(A) Control (CK), basal diet; (B) EO1, essential oils 200 g/t; (C) EO2, essential oils 600 g/t; (D) EO3, essential oils 1200 g/t; (E) EO1+AG, EO 200 g/t + florfenicol (0.15 g/kg, days 7–21); (F) EO3+AG, EO 1200 g/t + florfenicol (0.15 g/kg, days 7–21); (G) AG, florfenicol (0.15 g/kg, days 7–21).
